# A 3D Printable Thermal Energy Storage Crystalline Gel Using Mask-Projection Stereolithography

**DOI:** 10.3390/polym10101117

**Published:** 2018-10-09

**Authors:** Yuchen Mao, Takuya Miyazaki, Kohei Sakai, Jin Gong, Meifang Zhu, Hiroshi Ito

**Affiliations:** 1 Department of Polymer Science and Engineering, Graduate School of Organic Materials Science, Yamagata University, 4-3-16 Jonan, Yonezawa, Yamagata 992-8510, Japan; maoyc91@gmail.com; 2 State Key Laboratory for Modification of Chemical Fibers and Polymer Materials, College of Materials Science and Engineering, Donghua University, Shanghai 201620, China; 3 Department of Mechanical Systems Engineering, Graduate School of Science and Engineering, Yamagata University, Yonezawa, Yamagata 992-8510, Japan; trw55890@st.yamagata-u.ac.jp (T.M.); tmf62483@st.yamagata-u.ac.jp (K.S.)

**Keywords:** crystalline gel, 3D printing, mask-projection stereolithography, thermal energy storage, phase change material, thermoregulation

## Abstract

Most of the phase change materials (PCMs) have been limited to use as functional additions or sealed in containers, and extra auxiliary equipment or supporting matrix is needed. The emergence of 3D printing technique has dramatically advanced the developments of materials and simplified production processes. This study focuses on a novel strategy to model thermal energy storage crystalline gels with three-dimensional architecture directly from liquid resin without supporting materials through light-induced polymerization 3D printing technique. A mask-projection stereolithography printer was used to measure the 3D printing test, and the printable characters of crystalline thermal energy storage P(SA-DMAA) gels with different molar ratios were evaluated. For the P(SA-DMMA) gels with a small fraction of SA, the 3D fabrication was realized with higher printing precision both on milli- and micro- meter scales. As a comparison of 3D printed samples, P(SA-DMAA) gels made by other two methods, post-UV curing treatment after 3D printing and UV curing using conventional mold, were prepared. The 3D printed P(SA-DMAA) gels shown high crystallinity. Post-UV curing treatment was beneficial to full curing of 3D printed gels, but did not lead to the further improvement of the crystal structure to get higher crystallinity. The P(SA-DMAA) crystalline gel having the highest energy storage enthalpy was developed, which reached 69.6 J·g^−1^. Its good thermoregulation property in the temperature range from 25 to 40 °C was proved. The P(SA-DMAA) gels are feasible for practical applications as one kind of 3D printing material with thermal energy storage and thermoregulation functionality.

## 1. Introduction

Energy is the foundation of all creatures on the planet and essential for the development and evolution of mankind. Rapid worldwide development contributes to a huge demand for energy. Recovering waste heat and storage/release thermal energy are important for saving energy and reducing dependency on conventional fossil energy. Latent heat energy storage systems can use residual heat and store energy in highly efficient and environmentally friendly ways. Phase change materials (PCMs) are considered to be among the most reliable latent heat storage and thermoregulation materials, which work by absorbing or releasing the enthalpy of phase changes in certain temperature ranges. Now PCMs have been successfully utilized in aerospace and aviation, building, textile, preservation and environmental control [1,2,3,4]. Latent heat storage systems can be generally divided into two groups: compact and encapsulated [5]. In a compact system, PCMs are enclosed within a large container with an embedded heat exchanger, integrated with heat transfer fluid. Encapsulation technology is prominent for utilization of PCMs [6]. Micro or nano encapsulation is the process by which individual particles or droplets of solids or liquid materials (the core) are surrounded or coated with a continuous film of polymeric materials (the shell) [7,8]. However, PCMs are used as functional additions or sealed in containers, and extra auxiliary equipment or supporting materials are needed. If PCMs can be modeled with three-dimensional architecture, and processed in situ without supporting materials, it will open the possibility of PCMs for broader applications.

Since developed in the 1980s, three-dimensional (3D) printing, also known as additive manufacturing (AM), has been presented in art, architecture, tissue engineering, biomedical devices and manufacturing, such as rapid pattern making and rapid tooling using printing technologies [9,10,11,12]. 3D printing technology provides a possible method to manufacture objects with complex and precise structures, and unique physical properties for achieving optimal performance [13,14]. Through ingenious design, 3D printing subjects can improve initial properties and achieve more structure functions [15,16]. Fused decomposition modeling, selective laser sintering, stereolithography (SLA) and 3D plotting/direct-writing/bioprinting are the common 3D printing methods [16,17,18,19]. Above all, we are most interested in mask-projection SLA, which is an easy and quick 3D printing method using liquid ultraviolet (UV) curable photo-polymer and a UV laser to build solid objects by printing thin layers on top of each other [11,20]. The advantages of SLA are the ability to create complex shapes with internal architectures, ease of removal of unpolymerized resin and extremely high feature resolution (~1.2 μm) [11].

In our group, much of the work revolves around crystalline gels. We have developed crystalline gels with high toughness, high flexibility, and functions such as shape memory, humidity regulation, thermal expansion, and thermal energy storage, etc. [21,22,23,24,25]. These crystalline gels are synthesized by radical photo-polymerization that involves photo-reactive reagents having the property of curing from liquid to solid with light of sufficient energy. Note that the mask-projection SLA uses the same principle of photo-polymerization as the synthesis of crystalline gels. Therefore, we are interested in the 3D printing of crystalline gels in order to make three-dimensional objects directly from the reactive liquid resin.

**Figure 1:**
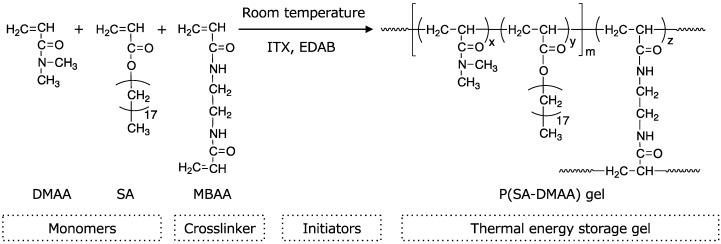
Synthesis scheme of the thermal energy storage crystalline P(SA-DMAA) gel. A light-induced random polymerization is processed between phase change monomer SA and another vinyl monomer DMAA with crosslinker MBAA at the aid of co-initiator system ITX-EDAB.

We designed a bulk photo-polymerization reaction system and combined it with 3D printing technique to synthesize and fabricate the energy storage crystalline gels at the same time on a 3D printer. The energy storage crystalline P(SA-DMAA) gels were synthesized by using stearyl acrylate (SA) and *N*,*N*-dimethylacrylamide (DMAA) as monomers, *N*,*N*′-methylenebisacrylamide (MBAA) as crosslinking agent, and 2-isopropylthioxanthone (ITX) and ethyl 4-dimethylaminobenzoate (EDAB) as co-initiation system. The synthesis scheme of P(SA-DMAA) gel is shown in Figure 1. The structure of SA consists of a vinyl group and a long alkane chain with 18 methylene units. The vinyl group has reactivity to form a polymer with the aid of photo initiators, while the long alkane chain promises the high crystallinity providing the thermal energy storage capacity [24]. The rapid curing time of acrylic monomers, which is normally within a range of seconds, promises the great potential of P(SA-DMAA) gel as a 3D printable material [26,27]. The crosslinker MBAA has two vinyl groups to conduct the radical reaction and to form crosslinking point between polymer chains to build up the three-dimensional mesh structure of gel. In this research, using a mask-projection stereolithography 3D printer, we discussed the 3D printing test and evaluated the printable characters of thermal energy storage crystalline P(SA-DMAA) gels.

## 2. Materials and Methods

### 2.1. Materials

SA and DMAA were purchased from Tokyo Chemical Industry Co., Ltd., Tokyo, Japan and used as monomers. Crosslinking agent of MBAA and photo initiators of ITX and EDAB were bought from Wako Pure Chemical Industries Ltd., Osaka, Japan. All the above reagents were used without further purification.

### 2.2. 3D Printing

**Figure 2:**
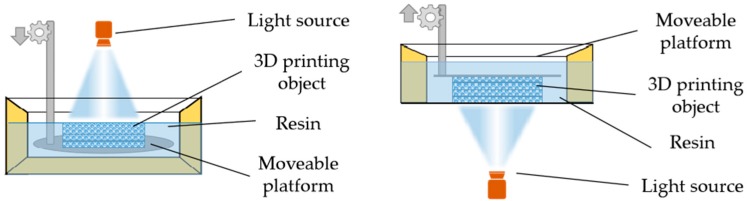
The schematics of mask-projection stereolithography for bottom-up system (**left**) and top-down system (**right**). The light source is scanning laser or digital light projection.

A mask-projection SLA printer normally consists of a light source, a movable platform for holding a printed object and a resin container. The light source also acts as a dynamic mask generator for obtaining diverse structures. A computer-controlled laser, an LED screen or a project can be used as a light source. Typically, there are two different working principles of mask-projection SLA, bottom-up system and top-down system. Figure 2 shows their working principles and schematics. The difference between bottom-up system and top-down system is the moving direction of the movable platform. In a bottom-up system, the holding platform goes down to reach the next position. On the contrary, in a top-down system, the holding platform moves upward to the next position. In both a bottom-up system or top-down system, the layer is cured one after another. After one layer is cured, the platform with the cured object moves to the next position, and then uncured liquid resin spreads over the previous layer. The following layer is now ready to be patterned. When object printing is finished, the liquid unpolymerized resin is removed. With a 3D printer, parameters including the power of a light source, exposure time and scanning speed, affect the layer thickness, accuracy of object and printing fidelity. The 3D printing speed can also be controlled by the chemical designs, monomer structures, formulation and the concentration of photo initiators [28,29,30,31]. Usually, post-curing in an UV oven is needed to convert unreacted groups, and to strengthen the structure.

**Figure 3:**
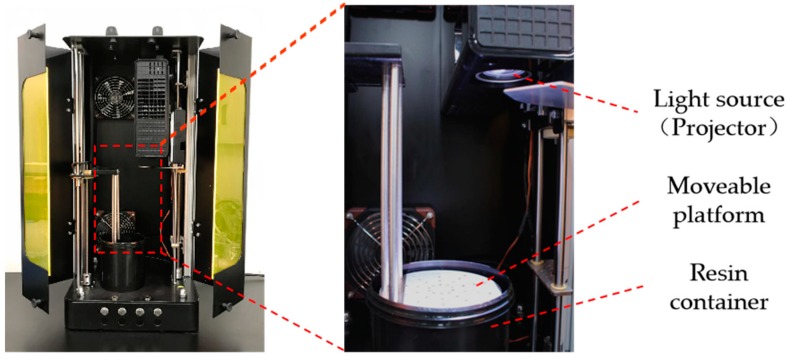
LumiForge, a mask-projection stereolithography 3D printer working from a bottom-up principle; its light source is provided by a projector.

We used a mask-projection SLA printer LumiForge made by Lumi industries, Italy (Figure 3) to conduct the 3D printing of the thermal energy storage P(SA-DMAA) gels. This printer is a commercial printer based on a top-down working principle. LumiForge consists of a UV–Vis light source, a resin container and a movable platform for holding a cured object mounted on a linear actuator. The building volume of LumiForge is 100 mm in diameter and 100 mm in height. LumiForge provides three optional Z slicing resolution (µm), including 100, 75 and 37.5. The light source is provided by a projector (Acer P1500 DLP Projector, Taoyuan, China) as a dynamic mask generator, which shows the image of each layer for the certain exposure time during the curing process. The maximum resolution of the projector is 1920 × 1080, ensuring precise structures and a great fidelity in printing. The wavelengths of the light source are in the visible light range, which is close to the working range of the ITX-EDAB co-initiation system. Additionally, the ITX-EDAB co-initiation system works quite efficiently under the visible light, which ensures short exposure time for each layer, promising a good printing performance. Thus, the oil-soluble ITX-EDAB initiation system is highly compatible with the monomers and crosslinker selected for this study.

**Figure 4:**
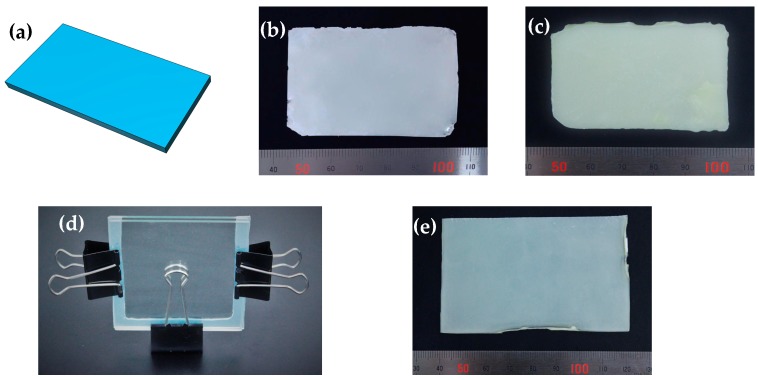
The 3D sheet model designed by 123D Design (**a**); P(SA-DMAA) gel sheet of 3DP 0.25 (printed on LumiForge) (**b**); Post-UV 3DP 0.25 (after post-UV treatment) (**c**); and Mold 0.25 (**e**); prepared using conventional mold (**d**).

The structural and functional test samples were 3D printed by adapting and optimizing the print parameters including layer thickness, layer exposure time, light source, and base layers, etc. The thermal energy storage crystalline P(SA-DMAA) gels were used as printing material. The uncured liquid gel solution, known as the printing ink, consists of monomers SA and DMAA, oil-soluble initiators ITX and EDAB, and crosslinking agent MBAA. ITX-EDAB co-initiation system shows high efficiency under UV–vis light. ITX, EDAB, and MBAA were dissolved in DMAA firstly, and then melted SA was added to prepare a homogeneous printing ink. The prepared printing ink was transferred into the resin container of LumiForge. The digital data of 3D model files were designed and edited using 123D Design open-source software (Autodesk Inc., San Rafael, CA, USA) to ensure a correct size for LumiForge. Specialized LumiCreator software (Lumi industries, Treviso, Italy) was used to control this 3D printer. After loading the digital datum of a 3D model in Stl file format, the Z slicing increment was set to 37.5 µm, which was the maximum slicing resolution for LumiForge. The first layer was cured on the surface of the movable platform. The platform would immerse a bit further into the resin before re-joining the correct position. The exposure time of the first 5 to 8 layers was lengthened to 45 s ensuring a perfect adherence of the object to the platform. After one layer cured, the platform moved downward to the next position, and another layer of uncured resin spread over the top layer. The exposure time for curing each layer was 30 s. The above processes repeated till the designed object was built up on LumiForge. Three printing inks with the molar ratios of SA to DMAA at 0.50:1.00, 0.33:1.00 and 0.25:1.00 were used to fabricate P(SA-DMAA) gel objects, named 3DP 0.50, 3DP 0.33 and 3DP 0.25, respectively. The 3D printed P(SA-DMAA) gel sheets were used for characterization. 3D data and printed gel sheets are shown in Figure 4a,b. In order to investigate the influence of post-UV curing treatment, those printed sheet samples treated by post-UV irradiation for 24 h at 30 °C (Figure 4c) marked as Post-UV 3DP 0.50, Post-UV 3DP 0.33 and Post-UV 3DP 0.25, respectively, were also prepared.

### 2.3. Preparation of Gel Sheets by Conventional Mold

As a comparison of 3D printed gel sheets, we synthesized three P(SA-DMAA) gel sheets as the same ingredients as 3DP 0.50, 3DP 0.33 and 3DP 0.25 by conventional mold (Figure 4d). The reactive gel liquid resin was injected into the mold with 1 mm thickness and cured under UV irradiation for 48 h at 30 °C. The prepared conventional P(SA-DMAA) gels were named Mold 0.50, Mold 0.33 and Mold 0.25, and the photograph of gel sheet for Mold 0.25 is shown in Figure 4e.

### 2.4. Characterization of 3D Printed P(SA-DMAA) Gels

#### 2.4.1. Fourier Transform Infrared Spectrometer (FTIR)

Chemical structures of P(SA-DMAA) gels were evaluated using a Fourier transform infrared spectrometer (FT/IR-460 Plus, JASCO International Co., Ltd., Tokyo, Japan). Each spectrum was recorded with 32 scans in transmittance mode with a resolution of 0.5 cm^−1^ within the range of 600 to 3300 cm^−1^.

#### 2.4.2. Wide-Angle X-ray Scattering (WAXS)

To determine the crystallinity of P(SA-DMAA) gels, wide-angle X-ray scattering (WAXS) was performed on an X-ray diffractometer (Ultima IV, Rigaku Corporation, Akishima, Japan) with nickel-filtered Cu Kα radiation at a scanning rate of 5 °C·min^−1^. The degree of crystallinity (*W*_c_, %) was evaluated according to the following formula [32,33]:

(1) $$W_{c}=\frac{I_{c}}{I_{c}+I_{a}}\times 100\%$$

where *I*_c_ and *I*_a_ are the scattering intensities of crystalline region and amorphous region, respectively.

#### 2.4.3. Differential Scanning Calorimetry (DSC)

The thermal energy storage capacity and phase change behavior of the P(DMMA-SA) gels were investigated using a differential scanning calorimeter (DSC) (Q-2000, TA instruments Japan Inc., Tokyo, Japan) operating under a nitrogen flow. Samples of about 5 mg were heated to 75 °C at a heating rate of 5 °C·min^−1^ and held for 1 min to eliminate the thermal prehistory. Next, samples were cooled down to 0 °C at a cooling rate of 5 °C·min^−1^ and held for 1 min, and finally reheated up to 75 °C at the same rate. Premium hermetic pans (TA Instruments Tzero #901683.901) were used for the measurements.

#### 2.4.4. Infrared Thermography

Thermal storage and release properties of 3D printed crystalline P(SA-DMAA) gels were evaluated by an infrared thermal graphic camera (FLIR E6, FLIR Systems Inc., Wilsonville, OR, USA). The samples were heated up to 70 °C and then cooled down to 0 °C on a hot stage.

## 3. Results and Discussion

### 3.1. Printing Performance of Thermal Energy Storage Crystalline Gels

**Figure 5:**
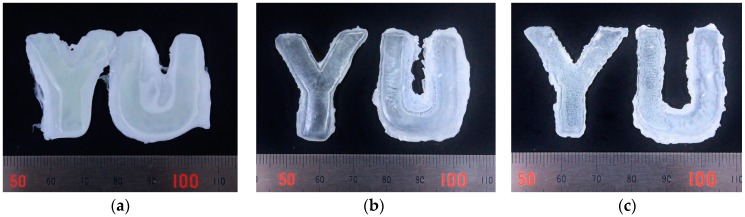
Characters “YU” printed on LumiForge under the same condition with different molar ratios of SA to DMAA at 0.50:1.00 (3DP 0.50) (**a**), 0.33:1.00 (3DP 0.33) (**b**) and 0.25:1.00 (3DP 0.25) (**c**). The printing performances improved with the decreasing molar fraction of SA.

Three thermal energy storage crystalline gels with different molar ratios of SA to DMAA at 0.50:1.00 (3DP 0.50), 0.33:1.00 (3DP 0.33) and 0.25:1.00 (3DP 0.25) were printed to evaluate the printing characters and performances. Characters “YU”, an abbreviation of Yamagata University, were printed on LumiForge under the same conditions. These 3DP objects were designed to be 2 mm in thickness. The thickness in Stl file was sliced by LumiCreator into 53 layers with maximum Z slicing resolution of 37.5 µm, so the printed thickness theoretically was about 1.98 mm. Figure 5 displays the printing results. As is shown, the printing test succeed for all the three P(SA-DMAA) gels with the average thickness about 1.74 mm, and the printing performances improved with the decreasing molar fraction of SA. The 3DP 0.50 object was printed with extra parts on the structure (Figure 5a), which was considered because the nearby liquid resin was also initiated and cured in the printing process. The object printed with 3DP 0.33 shows a clearer shape than 3DP 0.50 (Figure 5b). As shown in Figure 5c, when the molar fraction of SA reached 0.25, the 3DP 0.25 could be printed with a relatively higher resolution and a better fidelity than other two gels. The printed P(SA-DMAA) gel objects were flexible and ductile.

**Figure 6:**
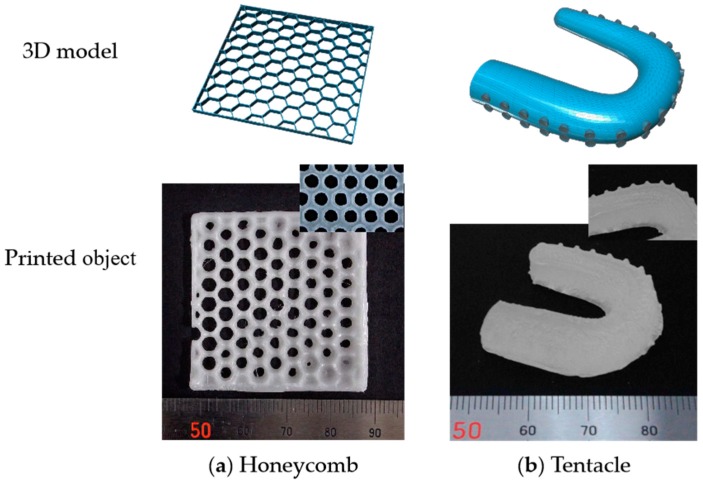
3D model data designed using the 123D Design software, and 3D objects fabricated on LumiForge with more complex shapes of honeycomb (**a**) and tentacle (**b**). The printed objects had high printing precision both on milli- and micro- meter scales.

We also designed objects with more complex shapes of honeycomb and tentacle using the 123D Design software, and printed them on LumiForge. In this case the P(SA-DMAA) gel with a small amount of SA, with the molar ratio of SA to DMAA at 0.25:1.00 was used. As shown in Figure 6, the printing tests were successful and the printed objects had a good printing fidelity. The manufactured honeycomb was flexible with the size of 40 × 40 × 2 mm, which is almost the same as the designed size. In addition, the hexagonal structure near the center was relatively complete. The average inner diameter of the holes was about 2.80 mm, and the average thickness of the honeycomb skeleton was about 1.56 mm. In the case of the tentacle model, the micrometer-scale suckers on tentacle were also fabricated clearly, with the resolution about 0.93 mm. The above test results prove that 3D fabrication of P(SA-DMAA) gels with a higher printing precision both on milli- and micro- meter scales were feasible for practical applications.

### 3.2. Chemical Composition and Structure

**Figure 7:**
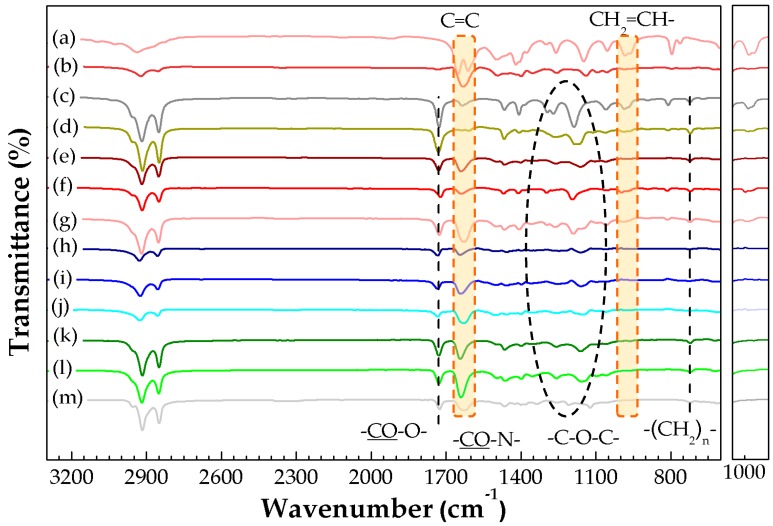
Fourier Transform Infrared Spectrometer (FTIR) spectra of P(SA-DMAA) gels and their partial enlargement around 1000 cm^−1^. (**a**) DMAA, (**b**) P(DMAA), (**c**) SA, (**d**) P(SA), (**e**) 3DP 0.50, (**f**) 3DP 0.33, (**g**) 3DP 0.25, (**h**) Post-UV 3DP 0.50, (**i**) Post-UV 3DP 0.33, (**j**) Post-UV 3DP 0.25, (**k**) Mold 0.50, (**l**) Mold 3DP 0.33, (**m**) Mold 3DP 0.25.

The chemical composition and structure of 3D printed P(SA-DMAA) gels were evaluated by FTIR spectroscopy. Figure 7 shows the infrared spectra of these gel samples. Thermal energy storage crystalline P(SA-DMAA) gels had a similar spectrum profile containing a series of characteristic absorption. All samples shown strong double intensive absorption peaks appearing at 2920 cm^−1^, 2850 cm^−1^ due to the alkyl C–H stretching vibrations of the methylene group. There was an absorption band at 1728 cm^−1^, which is attributed to the C=O stretching vibration of the ester group. The peaks at 1260, 1160 and 1130 cm^−1^, which can be assigned to the C–O stretching vibration of the ester group, were typical of acrylic ester. The infrared spectra also exhibited an absorption peak at 722 cm^−1^ corresponding to the in-plane rocking vibration of methylene groups belonging to the *n*-alkane side chain derived from SA. It was noted that two absorption bands near 1630 and 985 cm^−1^ in the infrared spectra had significant changes after polymerization. All P(SA-DMAA) gels shown the same strong absorption peak at 1630 cm^−1^ as P(DMAA), which is owing to the C=O stretching vibration of the tertiary amide. There were two characteristic absorption peaks for C=C. One was derived from SA at 1635 cm^−1^, and the other was derived from DMAA at 1610 cm^−1^. In addition, the characteristic absorption peak attributed to C–H stretching vibration of the end vinyl group presents at 985 cm^−1^. The two peaks at 1635 and 1610 cm^−1^ almost disappeared for 3D printed samples 3DP 0.50, 3DP 0.33 and 3DP 0.25 (Figure 7e–g), which indicates the radical reaction went well, and the P(SA-DMAA) gel objects could be synthesized and fabricated simultaneously on LumiForge. We noted that the weak absorption peak at 985 cm^−1^ for 3DP 0.33 and 3DP 0.25 disappeared after post-UV curing treatment (Figure 7i,j). The peak at 985 cm^−1^ also disappeared for the gel samples prepared by conventional mold under UV curing for 48 h (Figure 7k–m). This reveals post-UV curing treatment is advantageous to allow the 3D printed objects to be fully cured. We arrive at the same conclusions from the color of gel samples (Figure 4). The 3D printed gel sheet presents cream white (Figure 4b), the color of which is close to that of monomer SA. After 24 h post-UV curing treatment, the printed gel sheet (Figure 4c) shows milk yellow, which is the similar color to that of the gel sheet prepared by conventional mold (Figure 4e).

### 3.3. Crystalline Structure and Behavior

**Figure 8:**
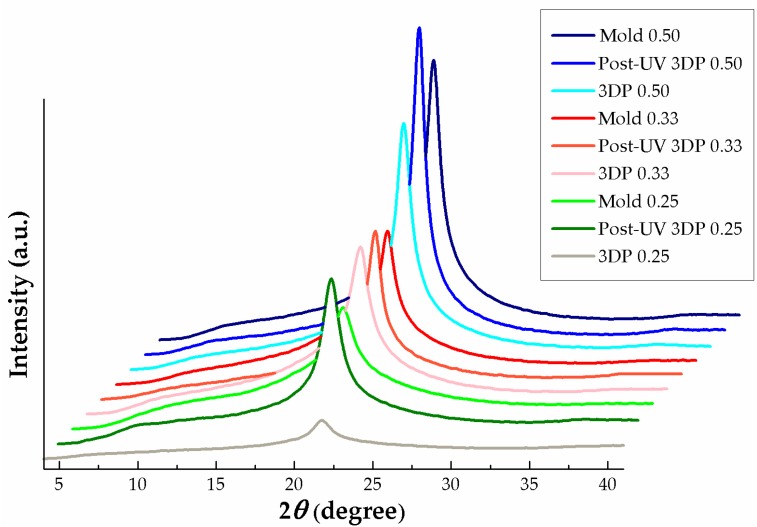
Wide-angle X-ray scattering (WAXS) patterns of thermal energy storage P(SA-DMAA) gels prepared with different methods and from different SA molar fractions. The high peak intensity represents the high crystallinity. Although no obvious influence of different methods on crystallinity was observed, the increasing amount of SA led to higher crystallinity.

The crystallization behavior of 3D printed thermal energy storage crystalline P(SA-DMAA) gels was investigated by WAXS analysis. WAXS patterns of 3DP gels along with post-UV curing treatment samples and conventional UV mold curing ones are shown in Figure 8. From the viewpoint of SA amount, it was found that the peak intensity increases with the increasing amount of SA. The high peak intensity and sharpness means the high crystallinity. Accordingly, the P(SA-DMAA) gels with the highest SA amount, 3DP 0.50, Post-UV 3DP 0.50 and Mold 0.50 shown the sharpest and strongest-intensity diffraction peak at 21.74°, indicating the highest crystallinity. The degree of crystallinity (*W*_c_) was estimated from the areas under the curves of the crystal region and amorphous region [25,32,34], and the values of *W*_c_ for all P(SA-DMAA) gels are listed in Table 1. The *W*_c_ of 3DP 0.50, Post-UV 3DP 0.50 and Mold 0.50 were around 40%, the highest among all P(SA-DMAA) gels, the main reason being that the addition of crystalline SA contributes to the polymer chain packing closely in a crystalline fashion, which increases the *W*_c_ of P(SA-DMAA) gels. The highest crystallinity and *W*_c_ promises a great potential for thermal energy storage. On the other hand, from the viewpoint of preparation method, although the 3DP and post-UV 3DP samples show a little higher crystallinity than samples prepared by conventional mold, no obvious influence of methods on crystallinity was observed. The result suggests that the post-UV curing treatment is beneficial to the further reaction, but not able to affect the further improvement of crystal structure to get higher crystallinity.

### 3.4. Energy Storage Capacity

**Figure 9:**
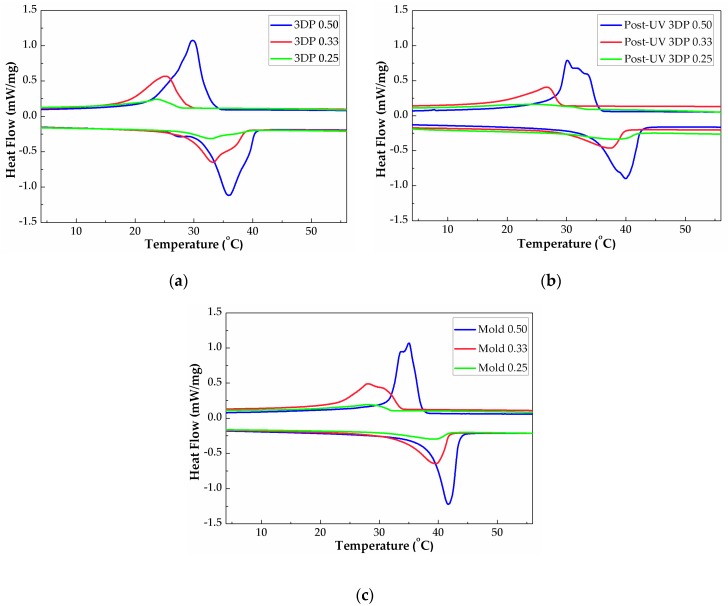
Differential Scanning Calorimetry (DSC) curves of thermal energy storage crystalline P(SA-DMAA) gels prepared from 3D printing (**a**), 3D printing with post-UV curing treatment (**b**), and curing with conventional UV mold (**c**).

Thermal energy storage/release behavior, phase change temperatures and their ranges, and phase change enthalpies are important parameters determining the thermoregulation capacities of phase change materials. Thermal energy storage capacity of crystalline P(SA-DMAA) gel was investigated through the DSC test. The DSC curves of gel samples, including 3DP, post-UV curing treatment and conventional UV mold curing gels, are shown in Figure 9. Their phase transition temperatures and phase change enthalpies measured by DSC are summarized and listed in Table 2. Under all the preparation methods, with the increasing amount of SA, the crystallization temperature (*T*_c_), the enthalpy of crystallization process (Δ*H*_c_), the melting temperature (*T*_m_), and the enthalpy of melting process (Δ*H*_m_) increases. That is, by increasing the amount of SA, energy storage capacity of P(SA-DMAA) gels were effectively improved. The increasing amount of crystalline SA leads to the amount of crystalline alkane side chains, which form better side-chain crystal regions for generating stronger crystalline/melting peaks and higher phase change enthalpies. Naturally, we can draw the conclusion that the increasing of SA contributes to better a thermal energy storage capacity.

P(SA-DMAA) crystalline gels with molar fraction of SA at 0.50 had the highest energy storage capacity in higher temperature ranges than other samples, reached over 69 J·g^−1^. It is noted that Mold 0.50 and Post-UV 3DP 0.50 presented a crystallization peak accompanied by one and two shoulders, whereas a single endothermic peak corresponding to the phase change of fusion. Many investigations on the crystallization behavior of *n*-alkane indicate that there are metastable rotator phases with transformation temperature in a few degrees above the crystallization temperature before the isotropic liquid completely converted into crystalline solid [35]. Rotator phases pass through one or more than one rotator phases between isotropic phase and crystal phase, due to the gradual breakdown of orientation order. These transitions between different phases correspond to the breaking of symmetry [36]. Such phase transition causes a multi-modal crystallization behavior for P(SA-DMAA) gels.

The phase transition behavior of 3DP P(SA-DMAA) gels were distinctive from conventional UV mold curing gels and post-UV curing treatment samples. At the same molar fraction of SA, the phase transition temperatures and phase change enthalpies of 3D printed gels are the highest among all P(SA-DMAA) gels prepared by the three methods. The post-UV curing treatment makes the melting behavior closer to samples made by conventional UV mold method. After post-UV curing treatment, phase transition temperatures of these 3D printed gels increased by about 2 °C, and their phase change enthalpy shown a decreased tendency. The difference in phase change behavior of 3D printed samples with and without post-UV curing treatment indicated that the post-UV curing treatment improved the further progress of polymerization and crosslinking reaction [31,37]. The higher Δ*H*_c_ and Δ*H*_m_ for 3DP 0.50 may partly derive from that of not-fully-reacted SA, as can be evidenced by FTIR results (Figure 7).

**Figure 10:**
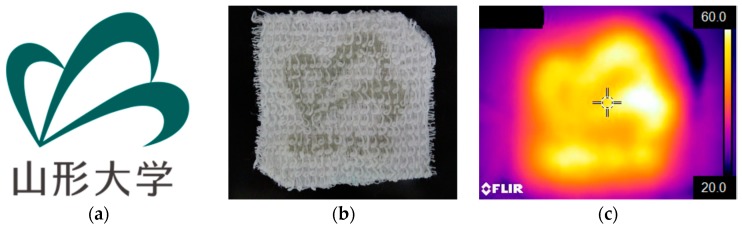
The logo of Yamagata University (**a**), the printed logo on cotton fabric using P(SA-DMAA) gel (3DP 0.25) as printing material on LumiForge (**b**), and its infrared thermal picture supplied by an infrared thermal graphic camera shown thermal energy storage capacity (**c**).

**Figure 11:**
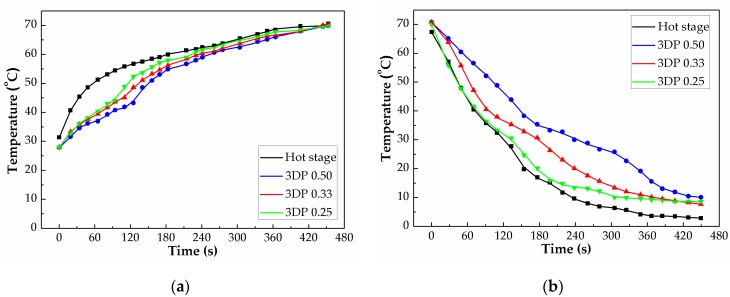
Temperature change as a function of time detected by an infrared thermal graphic camera for 3D printed crystalline P(SA-DMAA) gels in one heating (**a**) and cooling (**b**) cycle. A buffer zone and the temperature differences between 3DP gel samples and hot stage were clearly observed.

To further evaluate the thermal energy storage capacity of P(SA-DMAA) gel, the logo of Yamagata University (Figure 10a) was printed on cotton fabric using LumiForge (Figure 10b), and its infrared thermal pictures were detected to give a visual image of difference in temperature due to the thermal energy storage capacity (Figure 10c). The crystalline P(SA-DMAA) gels were successfully printed on cotton fabric; even the complex characters “山形大学” could be recognized. The thermoregulation properties of printed samples were measured on a hot stage. In the heating process, the temperature of the hot stage was set at 70, and 0 °C in the cooling process. The detected temperature changes with time in the heating and cooling process supplied by an infrared thermal graphic camera were plotted and given in Figure 11. A buffer zone and the temperature differences between 3DP gel samples and hot stage were clearly observed in one heating and cooling cycle. In the heating process, the temperatures of gels were lower than surroundings, and in the cooling process were higher, which gives a direct evidence for energy storage capacity of P(SA-DMAA) gels. Compared to 3DP 0.25, owing to the high phase change enthalpy, 3DP 0.33 and 3DP 0.50 prolonged the time taken to reach the setting point and slowed down the temperature changing rate, indicating a better energy storage capacity. In the temperature range of 30–40 °C for the heating process and 35–20 °C for the cooling process, 3D printed P(SA-DMAA) gels showed the thermoregulation property to store phase change energy. In addition, noted that 3DP 0.50 provided a thermoregulation property approximately 4 °C higher than 3DP 0.33, owing to the higher molar fraction of SA, and higher phase transition temperature. These results are consistent with the DSC results mentioned above.

## 4. Conclusions

A mask-projection SLA printer—LumiForge—working from a bottom-up principle, was chosen to print gels with the characteristic of easily control and simple production process. Three energy storage crystalline P(SA-DMAA) gels with the molar ratios of SA to DMAA at 0.50:1.00, 0.33:1.00 and 0.25:1.00 were used as 3D printing materials. The objects with three-dimensional architecture were synthesized and fabricated directly from liquid resin on LumiForge. By optimizing the 3D printing conditions, including layer thickness, chemical designs, formulation and the concentration of photo initiators, all P(SA-DMAA) gels were 3D printed successfully. With the decreasing molar fraction of SA, the printing resolution and fidelity became better. For the P(SA-DMMA) gel with a small fraction of SA at 0.25, the 3D fabrication could be realized with higher printing precision both on milli- and micro-meter scales. As a comparison of 3D printed samples, P(SA-DMAA) gels made by other two methods, post-UV curing treatment after 3D printing and UV curing using conventional mold, were prepared. The 3D printed P(SA-DMAA) gels shown high crystallinity, ensuring their good thermal energy storage capacity. Post-UV curing treatment for 24 h was beneficial to 3D printed gels fully cured, but was not able to affect the further improvement of crystal structure to achieve higher crystallinity. By increasing the molar fraction of SA, energy storage capacity was effectively improved, due to the increasing amount of crystalline *n*-alkane side chains, which is advantageous to form side-chain crystal regions for generating stronger crystalline/melting peaks and higher phase change enthalpies. P(SA-DMAA) crystalline gels with molar fraction of SA at 0.50 had the highest energy storage enthalpy, which reached 69.6 J·g^−1^. The infrared thermal testing results proved 3D printed crystalline P(SA-DMAA) gels provided a thermoregulation property in the temperature range from 25 to 40 °C.

We confirmed that the 3D printable P(SA-DMAA) gels had good thermal energy storage capacity, and their 3D printing were successful. The objects were of complex 3D structure, and the designed patterns on cotton fabric were printed with a high precision. The P(SA-DMAA) gels are feasible for practical applications as one kind of 3D printing material with thermal energy storage and thermoregulation functionality. We expect the 3D printable P(SA-DMAA) gels can be used to create a novel thermal energy storage device that is both complex in structure and wearable.

## Figures and Tables

**Table 1:** The degree of crystallinity (*W*_c_) evaluated from WAXS patterns for P(SA-DMAA) gels.

Sample	*W*_c_ (%)	Sample	*W*_c_ (%)	Sample	*W*_c_ (%)
Mold 0.50	39.16	3DP 0.50	41.24	Post-UV 3DP 0.50	41.52
Mold 0.33	27.61	3DP 0.33	28.79	Post-UV 3DP 0.33	26.30
Mold 0.25	19.05	3DP 0.25	23.57	Post-UV 3DP 0.25	22.52

**Table 2:** Phase transition temperatures and phase change enthalpies measured by DSC for P(SA-DMAA) gels.

Sample	*T*_c_ (°C)	Δ*H*_c_ (J·g^−1^)	*T*_m_ (°C)	Δ*H*_m_ (J·g^−1^)
3DP 0.50	29.78	69.22	35.88	69.58
3DP 0.33	25.27	37.65	33.16	41.24
3DP 0.25	23.55	12.04	32.67	13.49
Post-UV 3DP 0.50	30.10	53.01	39.88	56.68
Post-UV 3DP 0.33	26.89	23.53	37.32	23.95
Post-UV 3DP 0.25	24.48	11.54	37.76	11.71
Mold 0.50	35.03	53.73	41.66	53.42
Mold 0.33	28.01	36.12	39.56	34.48
Mold 0.25	28.01	9.54	39.00	10.58

*T*_c_: crystallization temperature, Δ*H*_c_: enthalpy of crystallization process, *T*_m_: melting temperature, Δ*H*_m_: enthalpy of melting process.
